# Whole-genome sequences of two novel chitinolytic bacterial strains within the genera *Streptomyces* and *Pseudomonas*

**DOI:** 10.1128/mra.01375-25

**Published:** 2026-04-20

**Authors:** Christian Larocque, Renlin Xu, Mercy Akuma, Cyr L. Doumbou, James T. Tambong

**Affiliations:** 1 Agriculture and Agri-Food Canada 6337, Ottawa, Ontario, Canada; 2 Institut des Sciences de la Santé et de la Vie, Ottawa, Ontario, Canada; 3 University of Ottawa 6363, Ottawa, Canada; University of Southern California, Los Angeles, California, USA

**Keywords:** *Streptomyces*, *Pseudomonas*, chitinolytic bacteria

## Abstract

*Streptomyces* sp. CLCI03 and *Pseudomonas* sp. CLCA07 were isolated from soil suspensions cultured on chitin-amended Winogradsky medium. Whole-genome analyses of these two strains revealed genome sizes of 8.68 and 6.73 Mb with G + C contents of 71.75% and 58.53%, respectively, and suggest that these strains represent two putative novel species.

## ANNOUNCEMENT

Chitinolytic bacteria degrade the second most abundant polymer, chitin, using complex enzymatic systems, and the byproducts serve as their nutrient sources. Chitin-degrading enzymes have been extensively studied and exploited to produce a range of products, including biofungicides, against major fungal plant pathogens since chitin is the key skeletal component of their cell walls (1–5). In 2023, we initiated a study to isolate, identify, and characterize novel putative chitin-degrading bacteria useful for plant bioprotection. Serial dilutions (10^−1^ and 10^−2^) of 0.1:10 soil suspensions in saline (wt/vol) were prepared and plated on Winogradsky salts (6) amended with 1% (wt/vol) colloidal chitin and incubated at 30°C for 72 h. Single colonies of two distinct isolates (CLCI03 and CLCA07) with halo zones were obtained from soils collected from Ottawa (45°26′16.4″ N, 75°37′49.1″ W) and Casselman (45°19′07.4″ N, 75°05′36.0″ W), Canada, respectively.

Genomic DNA extractions from overnight cultures (28°C; nutrient broth [Sigma-Aldrich, Canada]) were done, as previously described (7), and the same DNA was used for both libraries. Illumina (Nextera shotgun DNA kit) and Nanopore (SQK-LSK114, with size selection of ≥3 kb) genome sequencing libraries were constructed, as recommended by the manufacturers. Paired‐end (2 × 150 bp) raw reads were generated using the Illumina NovaSeq 6000 platform (Génome Québec, Montréal, Canada). Long reads were obtained in-house on MinION Mk1C using FLO-MIN114 v.R10.4.1 flow cell with Dorado v7.9.8 for base calling (Oxford Nanopore). The numbers of reads generated are given in Table 1. Trimmomatic v0.39 (8) was used for quality control and trimming of paired-end reads. Quality filtering and trimming of raw long reads were done using Chopper v8.4.0 (9) . Hybrid *de novo* assemblies were performed using Flye v2.9.6 (10) and polished with pilon (11) using short reads and quality checked by QUAST v5.2.0 (12), as implemented in the Bacterial and Viral Bioinformatics Resource Center pipeline (13, 14). Contigs less than 300 bp were discarded.

**TABLE 1:** Statistics of hybrid whole-genome sequencing, assembly, and annotation of strains *Streptomyces* sp. CLCI03 and *Pseudomonas* sp. CLCA07^*a*^

Parameters	Bacterial strain	
	CLCI03	CLCA07
Genus-level/species-level identification^*a*^	*Streptomyces* sp.	*Pseudomonas* sp.
Number of short paired reads (151 bp)	132,345,158	92,473,384
Number of long reads (average length, bp)	56,036 (7,482.5 bp)	33,842 (10,536.1 bp)
Average short-read coverage	294×	283×
Average long-read coverage (N_50_)	47× (8.83 kb)	52× (13.05 kb)
Chromosomal DNA size (bp)	8,678,721^*c*^	6,733,244^*d*^
Contig count	4	1
N_50_ (bp)	8,678,721	6,733,244
Contamination (%)^*b*^	0.70	4
G + C content (%)	71.75	58.53
Completeness (%)^*b*^	100	98.3
Coarse consistency (%)	98.9	99.0
Fine consistency (%)	95.7	97.0
Total CDSs^*e*^	7,878	6,072
CDSs with protein	7,690	5,928
tRNA	90	71
ncRNAs	3	4
rRNA (5S, 16S, and 23S)	21	22

^*a*^ Based on genome-based DNA-DNA hybridization and average nucleotide identity values, these strains represent putative novel bacterial lineages yet to be described.

^*b*^ The completeness and contamination were calculated using CheckM v1.0.18, as implemented in the Bacterial and Viral Bioinformatics Resource Center pipeline (14).

^*c*^ Contigs of CLCI03 are linear.

^*d*^ The genome sequence of CLCA07 is circular and complete.

^*e*^ CDS, coding sequence.

**Fig 1:**
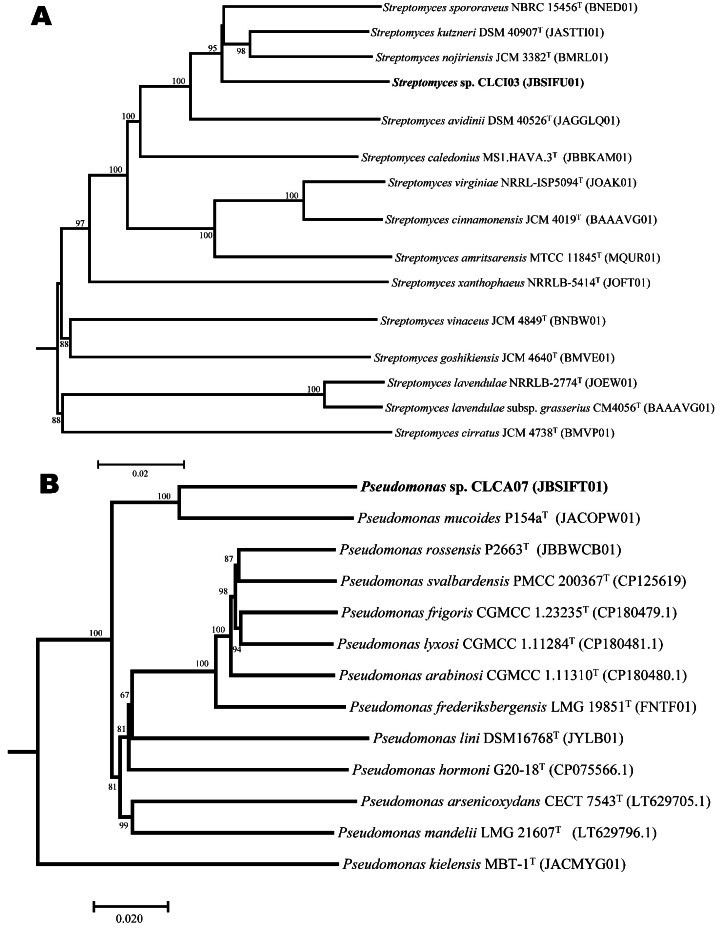
Phylogenomic trees inferred by the TYGS pipeline (20, 21) using the Genome BLAST Distance Phylogeny (GBDP) approach showing *Streptomyces* sp. CLG03 (**A**) and *Pseudomonas* sp. CLG04 (**B**) as putative novel species. The numbers above branches are GBDP pseudo-bootstrap support values of >60% from 100 replications, with an average branch support of 89.4%.

Total sizes of the draft genome sequences are 8,678,721 bp (linear) and 6,733,244 bp (circular and complete) for strains CLCI03 and CLCA07, respectively. NCBI Prokaryotic Genome Annotation Pipeline (15) annotated 7,878 and 6,072 coding sequences for CLCI03 and CLCA07, respectively. tRNAscan-SE v2.0.12 (16) detected 90 and 71 tRNAs for CLCI03 and CLCA07, respectively. Seven copies of rRNA operons (5S, 16S, and 23S) were predicted in CLCI03 and CLCA07, respectively, using Barrnap v0.9 (17), with the latter having an additional 5S rRNA. Other basic genome statistics, including N_50_ and G + C content, are shown in Table 1. Unless otherwise stated, default parameters were used for all software. Genome-based *in silico* DNA-DNA hybridization (dDDH (18)) and average nucleotide identity (ANI [19]) analyses could not taxonomically assign either strain to known species within the *Streptomyces* and *Pseudomonas* genera. The computed dDDH and ANI values were below the species delineation cut-off values of 70% and 95%–96%, respectively. The closest known species to strain CLCI03 is *Streptomyces nojiriensis* JCM 3382^T^ with 48.7% dDDH relatedness value, while strain CLCA07 exhibited the highest dDDH value of 42.5% with *Pseudomonas mucoides* P154a^T^. Phylogenomic analyses (20, 21) confirmed that these strains represent potential two novel lineages yet to be described (Fig. 1).

## Data Availability

This whole genome project has been deposited in GenBank under project number PRJNA1357910; BioSample numbers SAMN53096257 and SAMN53096258; and accession numbers JBSIFU000000000 and JBSIFT000000000 for strains CLCI03 and CLCA07, respectively. The Illumina and Nanopore raw sequence reads were deposited in the Sequence Read Archive database under accession numbers SRR36114699 and SRR36114698 for CLCI03, and SRR36114697 and SRR36114696 for CLCA07, respectively.

## References

[1] Itoh T, Kimoto H. 2019. Bacterial chitinase system as a model of chitin biodegradation. Adv Exp Med Biol 1142:131–151. doi:10.1007/978-981-13-7318-3_7 31102245

[2] Hamblin MR, Elieh-Ali-Komi D. 2016. Chitin and chitosan: production and application of versatile biomedical nanomaterials. Intern J. Advanced Research 4:411–427. PMC5094803 27819009

[3] Lenardon MD, Munro CA, Gow NAR. 2010. Chitin synthesis and fungal pathogenesis. Curr Opin Microbiol 13:416–423. doi:10.1016/j.mib.2010.05.002 20561815 PMC2923753

[4] Gong Z, Zhang S, Liu J. 2023. Recent advances in chitin biosynthesis associated with the morphology and secondary metabolite synthesis of filamentous fungi in submerged fermentation. JoF 9:205. doi:10.3390/jof9020205 36836319 PMC9967639

[5] Mapuranga J, Chang J, Li H, Zhang Y, Li R, Song L, Zhang N, Yang W. 2023. The molecular structure, biological roles, and inhibition of plant pathogenic fungal chitin deacetylases. Front Plant Sci 14:1335646. doi:10.3389/fpls.2023.1335646 38264029 PMC10803567

[6] Dabek-szreniawska M, Hattori T. 1981. Winogradsky’s salts solution as a diluting medium for plate count of oligotrophic bacteria in soil. J Gen Appl Microbiol 27:517–518. doi:10.2323/jgam.27.517

[7] Tambong JT, Xu R, Daayf F, Brière S, Bilodeau GJ, Tropiano R, Hartke A, Reid LM, Cott M, Cote T, Agarkova I. 2016. Genome analysis and development of a multiplex TaqMan real-time PCR for specific identification and detection of Clavibacter michiganensis subsp. nebraskensis. Phytopathology 106:1473–1485. doi:10.1094/PHYTO-05-16-0188-R 27452898

[8] Bolger AM, Lohse M, Usadel B. 2014. Trimmomatic: a flexible trimmer for Illumina sequence data. Bioinformatics 30:2114–2120. doi:10.1093/bioinformatics/btu170 24695404 PMC4103590

[9] De Coster W, Rademakers R. 2023. NanoPack2: population-scale evaluation of long-read sequencing data. Bioinformatics 39. doi:10.1093/bioinformatics/btad311 PMC10196664 37171891

[10] Kolmogorov M, Yuan J, Lin Y, Pevzner PA. 2019. Assembly of long, error-prone reads using repeat graphs. Nat Biotechnol 37:540–546. doi:10.1038/s41587-019-0072-8 30936562

[11] Walker BJ, Abeel T, Shea T, Priest M, Abouelliel A, Sakthikumar S, Cuomo CA, Zeng Q, Wortman J, Young SK, Earl AM. 2014. Pilon: an integrated tool for comprehensive microbial variant detection and genome assembly improvement. PLoS One 9:e112963. doi:10.1371/journal.pone.0112963 25409509 PMC4237348

[12] Gurevich A, Saveliev V, Vyahhi N, Tesler G. 2013. QUAST: quality assessment tool for genome assemblies. Bioinformatics 29:1072–1075. doi:10.1093/bioinformatics/btt086 23422339 PMC3624806

[13] Wattam AR, Davis JJ, Assaf R, Boisvert S, Brettin T, Bun C, Conrad N, Dietrich EM, Disz T, Gabbard JL, et al.. 2017. Improvements to PATRIC, the all-bacterial Bioinformatics Database and Analysis Resource Center. Nucleic Acids Res 45:D535–D542. doi:10.1093/nar/gkw1017 27899627 PMC5210524

[14] Olson RD, Assaf R, Brettin T, Conrad N, Cucinell C, Davis JJ, Dempsey DM, Dickerman A, Dietrich EM, Kenyon RW, et al.. 2023. Introducing the Bacterial and Viral Bioinformatics Resource Center (BV-BRC): a resource combining PATRIC, IRD and ViPR. Nucleic Acids Res 51:D678–D689. doi:10.1093/nar/gkac1003 36350631 PMC9825582

[15] Tatusova T, DiCuccio M, Badretdin A, Chetvernin V, Nawrocki EP, Zaslavsky L, Lomsadze A, Pruitt KD, Borodovsky M, Ostell J. 2016. NCBI prokaryotic genome annotation pipeline. Nucleic Acids Res 44:6614–6624. doi:10.1093/nar/gkw569 27342282 PMC5001611

[16] Chan PP, Lin BY, Mak AJ, Lowe TM. 2021. tRNAscan-SE 2.0: improved detection and functional classification of transfer RNA genes. Nucleic Acids Res 49:9077–9096. doi:10.1093/nar/gkab688 34417604 PMC8450103

[17] Seemann T. 2013. Barrnap version 0.9. Available from: https://github.com/tseemann/barrnap

[18] Meier-Kolthoff Jan P, Auch AF, Klenk H-P, Göker M. 2013. Genome sequence-based species delimitation with confidence intervals and improved distance functions. BMC Bioinformatics 14:60. doi:10.1186/1471-2105-14-60 23432962 PMC3665452

[19] Jain C, Rodriguez-R LM, Phillippy AM, Konstantinidis KT, Aluru S. 2018. High throughput ANI analysis of 90K prokaryotic genomes reveals clear species boundaries. Nat Commun 9:5114. doi:10.1038/s41467-018-07641-9 30504855 PMC6269478

[20] Meier-Kolthoff J.P, Göker M. 2019. TYGS is an automated high-throughput platform for state-of-the-art genome-based taxonomy. Nat Commun 10:2182. doi:10.1038/s41467-019-10210-3 31097708 PMC6522516

[21] Meier-Kolthoff Jan P, Carbasse JS, Peinado-Olarte RL, Göker M. 2022. TYGS and LPSN: a database tandem for fast and reliable genome-based classification and nomenclature of prokaryotes. Nucleic Acids Res 50:D801–D807. doi:10.1093/nar/gkab902 34634793 PMC8728197

